# Stigmatization and bias in interpreting lichen sclerosus risk factors

**DOI:** 10.1097/JW9.0000000000000242

**Published:** 2025-12-05

**Authors:** Sierra R. Parkinson, Sarah B. Corley

**Affiliations:** a University of North Carolina at Chapel Hill School of Medicine, Chapel Hill, North Carolina; b Department of Dermatology, University of North Carolina, Chapel Hill, North Carolina

**Keywords:** autoimmune, lichen sclerosus, stigma, surveillance bias, vulvar lichen sclerosus

**What is known about this subject in regard to women and their families?**

- Lichen sclerosus (LS) is a chronic inflammatory mucocutaneous condition that commonly affects the anogenital area and can significantly impact quality of life, including sexual and emotional well-being.
- The diagnosis of LS may be delayed due to its nonspecific or asymptomatic presentation, its location in sensitive anatomical areas, and the psychosocial stigma often associated with vulvar conditions.
- While the exact pathogenesis of LS remains uncertain, there is strong evidence supporting a multifactorial origin involving immune dysregulation and genetic susceptibility.

**What is new from this article as messages for women and their families?**

- This article highlights how biases in diagnosis and research data (e.g., surveillance bias) can create misleading associations, which might unintentionally increase stigma or blame.
- Our response emphasizes the importance of discussing all potential causes of LS, including its autoimmune basis, to support a more accurate and less stigmatizing understanding of the condition.
- Diagnosis can be complicated by misclassification and overlapping entities.

## Dear Editors,

The recent article published by Singal et al.^1^ investigating the association between prior anogenital infections and subsequent diagnoses of lichen sclerosus (LS) raises important clinical considerations. However, we are concerned about potential misinterpretations that may arise, especially among lay readers and nonspecialist clinicians.

First, the potential for surveillance bias is not adequately addressed in the article. The possibility of this bias exists given women with documented sexually transmitted infections, specifically herpes simplex virus and human papilloma virus, are more likely to be engaged in regular healthcare and undergo vulvar examinations by gynecologists or dermatologists, increasing the likelihood of subsequent LS diagnoses. Given that LS may be asymptomatic,^2^ women not receiving such surveillance may remain undiagnosed. Therefore, the increased LS prevalence in patients with prior sexually transmitted infection diagnoses reported by Singal et al. may reflect patterns of care rather than a true pathophysiologic association. Although the authors acknowledge an inability to infer causality, we believe the mention of surveillance bias is critical.

Second, the article does not reference the autoimmune pathogenesis, which is central to our current understanding of vulvar lichen sclerosus.^3^ Failure to highlight the significant evidence supporting autoimmune etiology risks allows readers to assume that LS is directly caused by infections or, worse, spread through sexual contact. This leads to a particularly harmful misconception for a disease that already significantly impacts quality of life and carries substantial psychosocial stigma,^4,5^ potentially making it even more difficult for patients to seek care.

Finally, in clinical practice, we have observed cases where patient’s LS-related erosions were attributed to herpes simplex virus. More broadly, diagnostic ambiguity, including challenges in distinguishing between diagnoses such as vulvar lichen sclerosus and extragenital lichen sclerosus, may contribute to inflated associations when using retrospective datasets, such as TriNetX, where clinical differentiation is not feasible.

We appreciate that the authors clearly state the study does not establish causation and acknowledge the limitations of using retrospective data. Nonetheless, we believe greater emphasis on surveillance bias, diagnostic uncertainty and the autoimmune etiology of LS would have strengthened the data interpretation and reduce stigma and delays in care for patients affected by LS.

## Conflicts of interest

None.

## Funding

None.

## Study approval

The author(s) confirm that any aspect of the work covered in this manuscript that has involved human patients has been conducted with the ethical approval of all relevant bodies.

## Author contributions

SRP: Drafted the manuscript. SBC: Provided critical revisions and oversight. All authors approved the final version.

## References

[1] Singal A Curtis KL Lipner SR. Prior anogenital herpes and human papillomavirus infections are associated with increased risk of lichen sclerosus in a large retrospective cohort study. Int J Womens Dermatol 2025;11:e210. 40469715 10.1097/JW9.0000000000000210 PMC12136664

[2] Lee A Bradford J Fischer G. Long-term management of adult vulvar lichen sclerosus: a prospective cohort study of 507 women. JAMA Dermatol 2015;151:1061–7. 26070005 10.1001/jamadermatol.2015.0643

[3] Tran DA Tan X Macri CJ Goldstein AT Fu SW. Lichen sclerosus: an autoimmunopathogenic and genomic enigma with emerging genetic and immune targets. Int J Biol Sci 2019;15:1429–39. 31337973 10.7150/ijbs.34613 PMC6643151

[4] Galentine E Powers CM McAleer L Rivera Dixon S Mauskar MM. Hidden hurts: life with lichen sclerosus—a patient’s perspective. Br J Dermatol 2024;192:153–4. 39267204 10.1093/bjd/ljae357

[5] Rivera S Dykstra C Flood A Herbenick D DeMaria AL. “Worse than disappointing”: prediagnostic health care challenges of women with inflammatory vulvar dermatoses. J Low Genit Tract Dis 2022;26:53–9. 34928253 10.1097/LGT.0000000000000632

